# ﻿Amphibians of the largest inland Atlantic Forest fragment, Iguaçu National Park, Paraná State, southern Brazil

**DOI:** 10.3897/zookeys.1264.164796

**Published:** 2025-12-15

**Authors:** Emerson A. da Rosa, Rodrigo Lingnau, Michel V. Garey

**Affiliations:** 1 Programa de Pós- Graduação em Biodiversidade Neotropical – UNILA, Av. Tarquínio Joslin dos Santos, 1000, Polo Jardim Universitário, Foz do Iguaçu, PR, Brazil Laboratório de Ecologia de Metacomunidades Foz do Iguaçu Brazil; 2 Laboratório de Ecologia de Metacomunidades – LEMET, Av. Tarquínio Joslin dos Santos, 1000, Polo Jardim Universitário, Foz do Iguaçu, PR, Brazil Programa de Pós- Graduação em Biodiversidade Neotropical – UNILA São Miguel do Iguaçu Brazil; 3 Universidade Tecnológica Federal do Paraná – UTFPR, Rua Gelindo João Folador, 2000, Bairro Novo Horizonte, Francisco Beltrão, PR, Brazil Universidade Tecnológica Federal do Paraná Francisco Beltrão Brazil

**Keywords:** Anurans, inventory, Mixed Ombrophilous Forest, Semideciduous Seasonal Forest, species list

## Abstract

The present study aimed to provide a species list of amphibians from Iguaçu National Park, the largest forest fragment of inland Atlantic Forest, located in the westernmost region of Paraná state, recognized as a UNESCO Natural Heritage Site. The park’s vegetation is predominantly composed of two types: Semideciduous Seasonal Forest (SSF) and Mixed Ombrophilous Forest (MOF). To compile the species list, primary data obtained through sampling adult anurans and tadpoles in 21 water bodies was used, complemented by search for historical records based on specimens deposited in scientific collections. 31 anuran species belonging to seven families were recorded, with the Hylidae being the richest, comprising 17 species. Two species, *Boana curupi* and *Physalaemus carrizorum*, represent the first records in the state of Paraná. The anuran species recorded in the Iguaçu National Park present nine reproductive modes, reflecting a diversity similar to that observed in other areas of Semideciduous Seasonal Forest. Twelve species were recorded exclusively in the SSF, eight species exclusively in MOF, and 11 species occurred in both vegetation types. The anuran assemblage of Iguaçu National Park is most similar to that Iguazú National Park in Argentina, with which it shares 24 species. Although the Iguaçu River is the only geographic barrier separating these two protected areas, the complementarity in amphibian species composition highlights the importance of conserving both parks for maintaining regional biodiversity.

## ﻿Introduction

Brazil harbors at least 1,188 amphibian species, including 1,144 anurans, 39 gymnophionans, and five caudatans (Segalla et al. 2021). The Atlantic Forest domain exhibits the highest amphibian species richness in the country (Campos et al. 2014; Rossa-Feres et al. 2017), with more than 625 anuran species recorded and more than 77% anuran species are endemic (Rossa-Feres et al. 2017). Given its exceptional species richness, combined with high endemism, and severe anthropogenic pressures, has led to the classification of the Atlantic Forest as one of the world’s biodiversity hotspots (Myers et al. 2000). Within the Atlantic Forest, areas of Dense Ombrophilous Forest contain the highest richness of anuran species (Rossa-Feres et al. 2017) and still remain as extensive forest remnants with relatively high connectivity (Rosa et al. 2021). In contrast, more degraded regions of the Atlantic Forest domain are dominated by phytophysiognomies that support lower species richness of anurans, such as the Semideciduous Seasonal Forest (SSF) and Mixed Ombrophilous Forest (MOF) (Rossa-Feres et al. 2017). In the western region of Paraná, originally covered by SSF with some patches of MOF, recent trends indicate increasing forest cover and reduced habitat isolation (Rosa et al. 2021). Despite these reforestation efforts, only 9% of the region remain forested (Instituto Água e Terra 2021), and most of these forest fragments are small and highly isolated (Rosa et al. 2021).

In the state of Paraná, surveys of anuran assemblages have been conducted primarily in the coastal region and the metropolitan area of Curitiba, where Dense Ombrophilous Forest is the dominant vegetation type (e.g., Conte and Rossa-Feres 2006; Armstrong and Conte 2010; Garey and Hartmann 2012), as well as the central-northern region, characterized by SSF and MOF (e.g., Conte and Machado 2005; Conte and Rossa-Feres 2007; Affonso and Delariva 2012; Nazaretti and Conte 2015). In contrast, the anuran assemblages in the MOF and SSF of western, southern, and southwestern regions of Paraná state remain understudied (Santos-Pereira et al. 2018). In the western region of Paraná state, only few remnants have been inventoried, including areas near Rio Guarani State Park in the municipality of Três Barras do Paraná (Bernarde and Machado 2001), the Bela Vista Biological Refuge in Foz do Iguaçu (Vicente-Ferreira et al. 2024), and some forest remnants and anthropogenic landscapes (farmlands and urban areas) in western of Paraná state (Leivas et al. 2018). Notably, Iguaçu National Park (INP) the largest forest fragment of inland Atlantic Forest, still lacks a systematic inventory of its amphibian assemblage (Santos-Pereira et al. 2018).

The INP is considered the largest and most important fragment of the Atlantic Forest in southern Brazil. It is the largest protected area in Paraná state and the second largest in the Atlantic Forest domain (Paviolo et al. 2016). The INP has been recognized as a UNESCO World Natural Heritage Site since 1986 (D’Oliveira et al. 2002). Part of INP is located in a transboundary region, sharing more than 60 kilometers of border with Iguazú National Park in Argentina, thus forming part of one of the most significant biological corridors in the south-central region of South America (Instituto Chico Mendes de Conservação da Biodiversidade 2018). Despite its ecological importance, knowledge of the INP anurans remains largely anecdotal in the scientific literature. Although some undergraduate theses and dissertations have addressed amphibians in INP (e.g., Jeziorny 2015; Nazaretti 2016), peer-reviewed articles include only sparse records: five species reported by Lutz (1973), two species by Heyer (1979, 1994), one species by Conte et al. (2009), and four species recorded by Leivas et al. (2018). This severe data deficiency is reflected in the INP Management Plan, which currently recognizes just 12 amphibian species for the entire park (Instituto Chico Mendes de Conservação da Biodiversidade 2018).

Given the lack of systematic inventories in the largest remaining forest fragment of the Atlantic Forest, and considering that such inventories are essential for providing baseline data to support the development of effective conservation and management strategies, this study aims to: (i) present a comprehensive list of amphibian species occurring in Iguaçu National Park; (ii) provide information on species’ natural history and habitat use; and (iii) compare the amphibian species composition in INP with assemblages from other regions that share the same vegetation types present in the park.

## ﻿Materials and methods

### ﻿Study area

**Figure 1.**
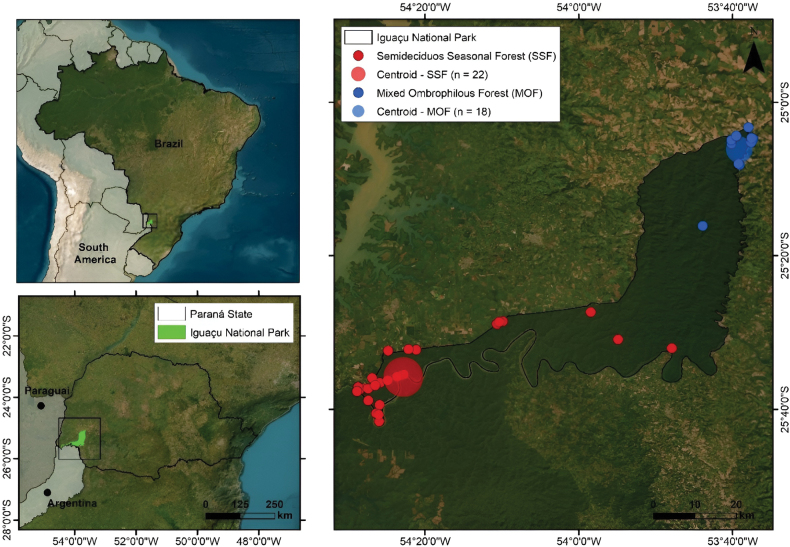
Geographic location of the study area: Iguaçu National Park, located in the extreme western region of the state of Paraná, Brazil. The centroids (larger circles) represent the average distances of the sampling points. Circle sizes vary according to anuran amphibian species richness and the number of sampling points within each phytophysiognomy: Semideciduous Seasonal Forest (SSF) and Mixed Ombrophilous Forest (MOF).

We conducted amphibians survey in the Iguaçu National Park, located in the western region of Paraná state, southern Brazil (Fig. 1). The INP is considered the main fragment of the Atlantic Forest in southern Brazil (Paviolo et al. 2016) and comprises two main vegetation types: Semideciduous Seasonal Forest (SSF) and Mixed Ombrophilous Forest (MOF) (Ribeiro et al. 2009). The vegetation type variation follows the altitudinal gradient across the park, which ranges from 100 to 750 meters above sea level, with MOF occurring at higher elevations and SSF at lower elevations (Cervi and Borgo 2007). The INP covers 185,262.5 hectares (Instituto Chico Mendes de Conservação da Biodiversidade 2018) and includes portions of six municipalities within its boundaries: Foz do Iguaçu, São Miguel do Iguaçu, Matelândia, Serranópolis do Iguaçu, Céu Azul, and Capanema. The surrounding region includes eight municipalities: Santa Terezinha de Itaipu, Santa Tereza do Oeste, Santa Lúcia, Lindoeste, Capitão Leônidas Marques, Medianeira, Vera Cruz do Oeste, and Ramilândia (Instituto Chico Mendes de Conservação da Biodiversidade 2018). The surrounding buffer area is occupied by intensive livestock farming and mechanized agriculture (Instituto Chico Mendes de Conservação da Biodiversidade 2018).

The climate in the INP is humid subtropical mesothermal (Cfa, under Köppen-Geiger classification) (Alvares et al. 2013). Mean annual temperature ranges between 20 °C and 22 °C, with January being the warmest month (mean 26.3 °C) and July the coldest (mean 16.6 °C). Annual precipitation ranging from 1600 to 1900 mm, distributed throughout the year without a distinct dry season. Monthly rainfall peaks in October, with an average of 186 mm, and reaches its minimum in July, with only 92 mm (Alvares et al. 2013).

### ﻿Sample design

To compile the amphibian species list for Iguaçu National Park, we combined data from field survey, scientific collections, and the literature. We only considered literature records when voucher specimens were deposited in a scientific collection. We obtained primary data through sampling lentic and lotic water bodies within the INP in the municipalities of Foz do Iguaçu and São Miguel do Iguaçu. We conducted three sampling campaigns between November 2023 and March 2024, maintaining a minimum interval of 30 days between them to maximize the likelihood of detecting species with different reproductive periods (e.g., Bolzan et al. 2019; Dubos et al. 2019).

We sampled amphibians, adult anurans and tadpoles, in 21 water bodies (18 lentic and three lotic). In each campaign, we surveyed each water body twice: once during the day for tadpole collection and once at night for adult anurans, with both diurnal and nocturnal sampling sessions lasting one hour. We captured tadpoles using dip nets with a mesh size of 3 mm (adjusted in diameter according to water body features). We euthanized collected tadpoles by immersion in a lidocaine solution and preserved them in a 1:1 solution of 10% formalin and 70% ethanol for later identification under a stereomicroscope. We sampled adult anurans at night using active search methods at breeding sites (Scott et al. 1994). We euthanized some adult individuals through topical lidocaine application, fixed them in 10% formalin, and then preserved in 70% ethanol. We deposited all collected individuals (tadpoles and adults) in Bertha Lutz Herpetological Collection at Unila (CA-Unila) (Suppl. material 1).

We obtained secondary data on amphibian occurrences in the INP from scientific collections and literature. We visited the scientific collections to verify and confirm specimen identifications at the Museu de História Natural Capão da Imbuia in Curitiba (**MNHCI-Herpeto**), Paraná, and the Bertha Lutz Herpetological Collection at the Universidade Federal da Integração Latino-Americana) in Foz do Iguaçu (**CA-Unila**), Paraná. Additionally, we conducted a systematic literature review using the search terms [“Amphibia” OR “Anura” OR “Gymnophiona”] AND [“Parque Nacional do Iguaçu” OR “Iguaçu National Park”] across Scopus, Web of Science, and Google Scholar platforms. We followed the taxonomic nomenclature proposed by the Amphibian Species of the World (Frost 2024).

For each species recorded in the INP, we compiled data on: (i) location of occurrence, (ii) phytophysiognomy, (iii) population status, (iv) reproductive mode, (v) habit, (vi) semaphoront, and (vii) habitat type. Location refers to the municipality where the individual was found. We classified phytophysiognomy as either SSF or MOF. We determined population status (increasing, decreasing, or stable) according to the IUCN assessments (International Union for Conservation of Nature and Natural Resources 2024). We categorized reproductive modes based on field observations and literature sources (i.e., Toledo et al. 2021), following the classification proposed by Haddad and Prado (2005). We defined the habit as the environment in which adult individuals typically occur: arboreal, rheophilic, terrestrial, cryptozoic, or fossorial (Toledo et al. 2021). The semaphoront refers to the life stage of the individuals observed, classified as either adult or tadpole. We classified habitat type based on the water body where individuals (tadpoles or adults) were recorded, classified as lotic (e.g., streams) or lentic (e.g., temporary and permanent ponds, and marsh), following field observations and literature sources (e.g., Toledo et al. 2021).

### ﻿Data analysis

We compared the anuran assemblage of Iguaçu National Park with 18 published inventories from Seasonal Forests (Deciduous or Semideciduous) and MOF using similarity index. We selected inventories based on two criteria: (i) the presence of similar phytophysiognomies and (ii) geographic distance less than 1000 km from the study area. The following inventories were included in the analysis: Refúgio Biológico Bela Vista (Vicente-Ferreira et al. 2024), Parque Estadual Rio Guarani (Bernarde and Machado 2001), Mata dos Godoy (Machado et al. 1999), São José dos Pinhais (Conte and Rossa-Feres 2006), Floresta Nacional de Piraí do Sul (Foerster and Conte 2018), Parque Municipal São Luís de Tolosa (Santos and Conte 2014), Fazenda Rio Grande (Conte and Rossa-Feres 2007), and Área de Proteção Ambiental da Serra da Esperança (Instituto Água e Terra 2009) in Paraná state; Estação Ecológica dos Caetetus (Brassaloti et al. 2010), Parque Estadual do Morro do Diabo (Santos et al. 2009), Floresta Estadual Edmundo Navarro de Andrade (Toledo et al. 2003) in São Paulo state; Parque Nacional da Serra da Bodoquena in Mato Grosso do Sul state (Uetanabaro et al. 2007); Parque Estadual Fritz Plaumann (Bastiani and Lucas 2013), Parque Nacional das Araucárias (Lucas and Marocco 2011), Floresta Nacional de Chapecó (Lucas and Fortes 2008) in Santa Catarina state; Parque Municipal de Sertão (Zanella et al. 2013), Parque Estadual do Papagaio Charão (Potrich et al. 2020) in Rio Grande do Sul state; Mbaracayú, Alto Paraná Department, in Paraguay (Cacciali et al. 2015); and Parque Nacional Iguazú, Misiones Province in Argentina (López and Garey 2021).

Due to methodological variations among inventories, we restricted our analysis to presence-absence data. We calculated community similarity using Jaccard index and performed a non-Metric Multidimensional Scaling (nMDS) to graphically evaluate the variation in the species composition among anuran inventories. All analyses were performed in the R software (R Core Team 2024) using the package ‘vegan’ (Oksanen et al. 2022).

## ﻿Results

**Figure 2.**
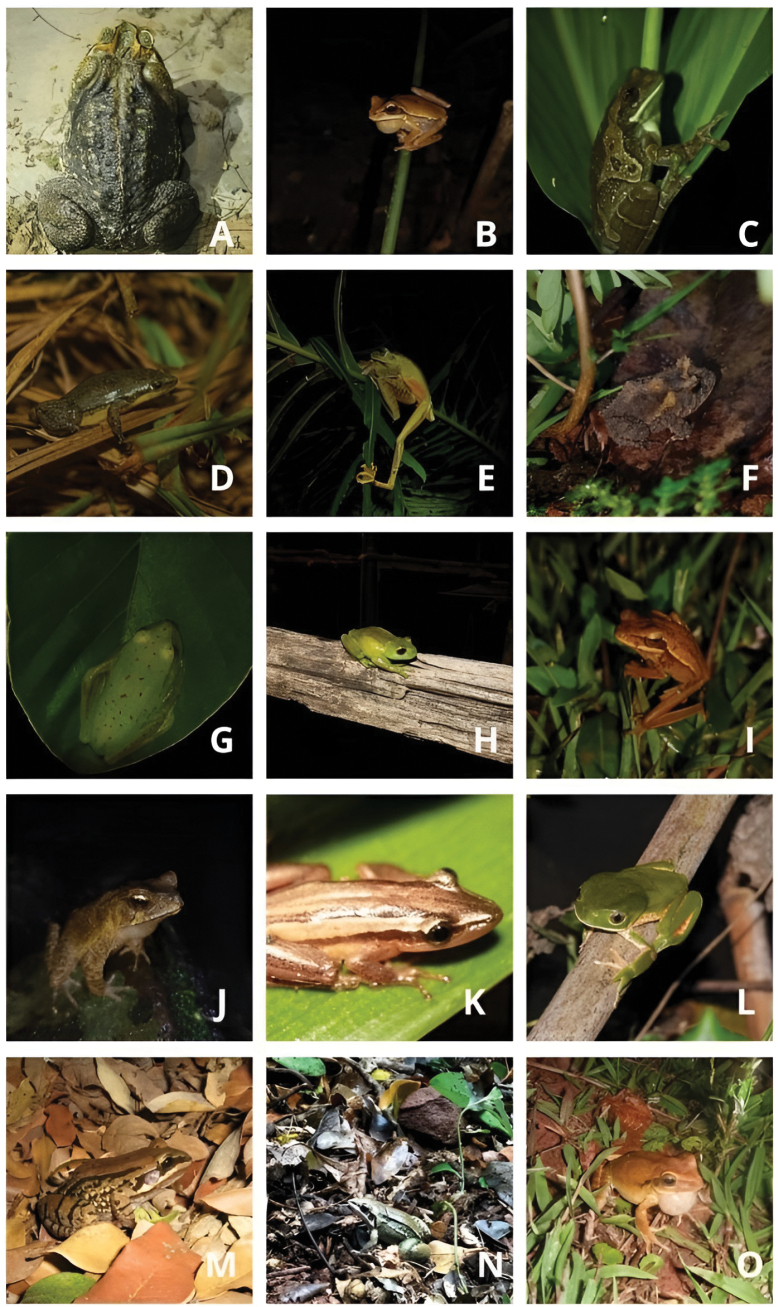
Anuran amphibians recorded in the Iguaçu National Park, western Paraná, southern Brazil. **A.***Rhinella diptycha*; **B.***Boana curupi*; **C.***Trachycephalus typhonius*; **D.***Elachistocleis bicolor*; **E.***Boana faber*; **F.***Proceratophrys avelinoi*; **G.***Boana punctata*; **H.***Aplastodiscus perviridis*; **I.***Boana caingua*; **J.***Rhinella ornata*; **K.***Scinax squalirostris*; **L.***Phyllomedusa tetraploidea*; **M.***Leptodactylus mystacinus*; **N.***Leptodactylus elenae*; **O.***Boana raniceps*.

**Figure 3.**
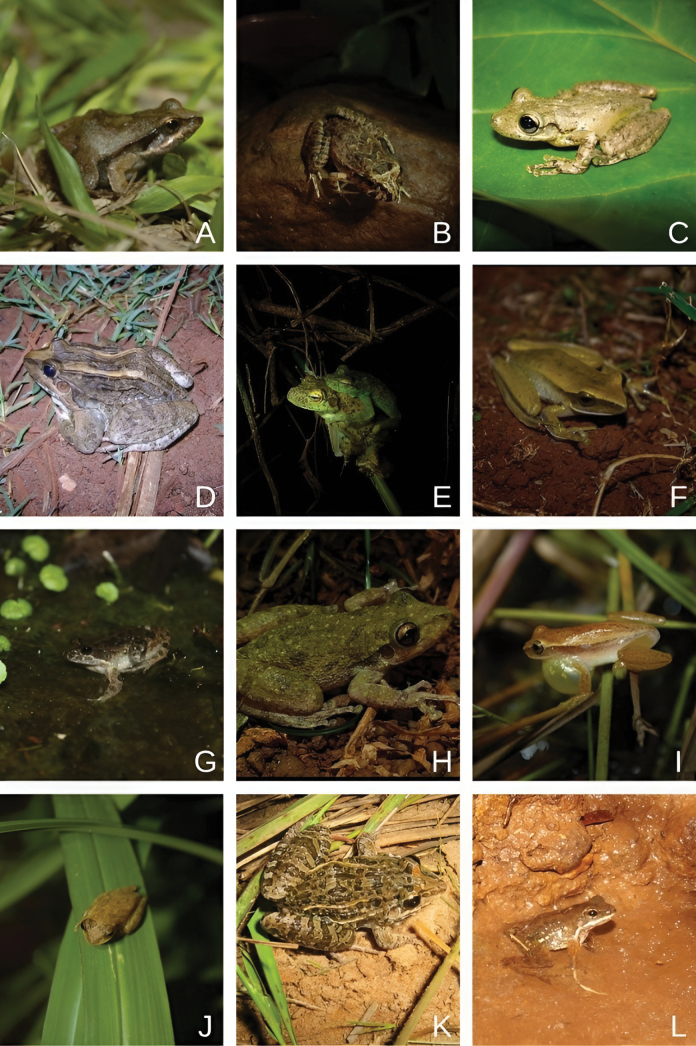
Anuran amphibians recorded in the Iguaçu National Park, western Paraná, southern Brazil. **A.***Physalaemus cuvieri*; **B.***Limnomedusa macroglossa*; **C.***Scinax perereca*; **D.***Leptodactylus luctator*; **E.***Itapotihyla langsdorffii*; **F.***Boana albopunctata*; **G.***Leptodactylus podicipinus*; **H.***Scinax fuscovarius*; **I.***Dendropsophus nanus*; **J.***Dendropsophus minutus*; **K.***Leptodactylus fuscus*; **L.***Crossodactylus schmidti*.

We recorded 31 anuran amphibian species in the Iguaçu National Park, including 19 species recorded during field sampling and 12 species from scientific collections. All recorded species belong to the Anura and are distributed across seven families: Hylidae (17 species, 54.8% of total), Leptodactylidae (*n* = 8, 25.8%), Bufonidae (*n* = 2, 6.5%), and four families with one species each (Alsodidae, Hylodidae, Microhylidae, and Odontophrynidae; 3.2% each) (Figs 2, 3).

**Figure 4.**
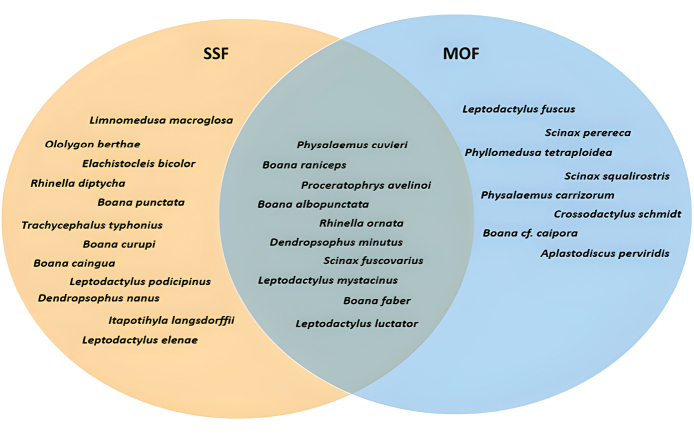
Venn diagram showing the distribution of anuran amphibian species between the two phytophysiognomies of the Iguaçu National Park: Semideciduous Seasonal Forest (SSF) and Mixed Ombrophilous Forest (MOF).

Among the municipalities within INP, Foz do Iguaçu had the highest species richness, with 22 species (71%) recorded. Céu Azul had 16 species (52%), followed by São Miguel do Iguaçu with ten species (32%). Only two species (6%) were recorded in Serranópolis do Iguaçu. Although Matelândia and Capanema are also part of the INP, we did not found records of amphibian collections from these areas. We recorded 11 species (39%) exclusively in the Semideciduous Seasonal Forest (SSF), eight species (26%) exclusively in the Mixed Ombrophilous Forest (MOF), and 11 species (36%) in both vegetation types within INP (Fig. 4).

The anuran amphibians recorded in the INP exhibit nine reproductive modes. The family Hylidae exhibited the highest richness of reproductive modes (six reproductive modes), followed by Leptodactylidae with three. All other families exhibited a single reproductive mode. All species of Alsodidae, Bufonidae, and Microhylidae recorded in the INP reproduce by lay eggs in still water, where tadpoles develop (mode 1). The only representative of Odontophrynidae lay eggs in flowing water, which hatch into exotrophic tadpoles (mode 2). The single Hylodidae species reproduced exhibits mode 3, in which eggs and larvae develop in submerged chambers, and exotrophic tadpoles inhabit streams. Species of Leptodactylidae exhibit three reproductive modes: floating foam nests in ponds, with exotrophic tadpoles developing in ponds (mode 11); foam nests in constructed basins, with tadpole development in ponds (mode 13); and foam nests in underground cavities, where flooding facilitated tadpole migration to ponds (mode 30). Hylidae species exhibited six reproductive modes: eggs deposited in natural or constructed basins that, upon flooding released exotrophic tadpoles into ponds or streams (mode 4); eggs deposited in constructed nests that, after flooding, allowed exotrophic tadpoles to develop in ponds and streams (mode 5); floating foam nests in ponds with tadpoles remaining in the same environment (mode 11); eggs hatching into exotrophic tadpoles in lentic water bodies (mode 24). Additionally, Hylidae included modes 1 and 2, as previously described.

**Figure 5.**
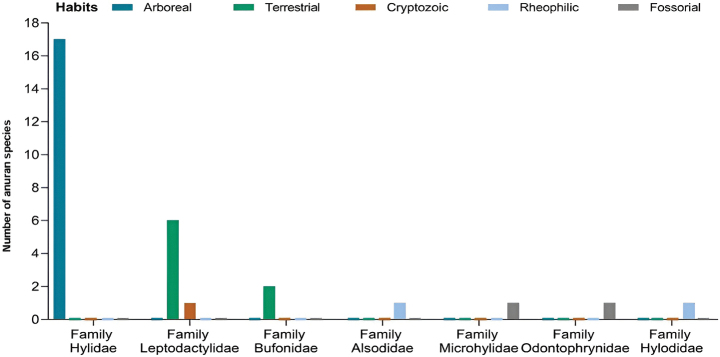
Chart illustrating the ecological habits of anuran amphibian species recorded per family in Iguaçu National Park, western region of the state of Paraná, Brazil.

Among the amphibians recorded in the INP, 17 species (55%) are arboreal, nine (29%) are terrestrial, two (6%) are rheophilic, two (6%) are cryptozoic, and one species (3%) is fossorial. All species in the family Hylidae are arboreal. Most Leptodactylidae species are terrestrial, with only one exception (*Leptodactylus podicipinus*) classified as cryptozoic. Both Bufonidae species are terrestrial. The species from the families Alsodidae and Hylodidae are rheophilic, while those from the Microhylidae and Odontophrynidae are fossorial and cryptozoic, respectively (Fig. 5). In terms of habitat type, 14 species (45%) occurred exclusively in lentic environments, six (19%) were restricted to lotic environments, and ten (33%) inhabited both habitat types.

**Figure 6.**
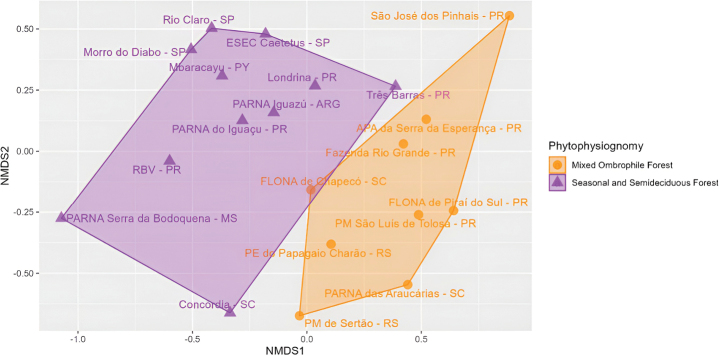
NMDS ordination plot based on the Jaccard Index (presence-absence data), showing the dissimilarity in anuran amphibian assemblages across areas of Seasonal Semideciduous Forest and Mixed Ombrophilous Forest. See materials and methods for information on other anuran assemblages.

The nMDS analysis (Fig. 6) revealed two distinct groups based on similarity in anuran assemblage composition: one composed by assemblages from SSF and the other from MOF. The anuran composition in the Iguaçu National Park showed greater similarity to that of the Iguaçu National Park in Misiones, Argentina, and the Refúgio Biológico Bela Vista in Foz do Iguaçu, western Paraná state, Brazil.

The complete list of anuran species recorded, including their taxonomic authorities and relevant ecological information, is presented in Table 1.

**Table 1.** Anuran amphibian species found in the Iguaçu National Park, westernmost state of Paraná, southern Brazil. Location: city to which the collection site belongs: FZ (Foz do Iguaçu); SMI (São Miguel do Iguaçu); SI (Serranópolis do Iguaçu); CA (Céu Azul). Vegetation formation: SSF (Seasonal Semideciduous Forest); MOF (Mixed Ombrophilous Forest), Population status: Increasing; Decreasing; Stable and Unknown. Reproductive mode: 1 = Eggs and exotrophic tadpoles in lentic water; 2 = Eggs and exotrophic tadpoles in lotic water; 3 = Eggs and initial larval stages in constructed underwater chambers, exotrophic tadpoles in streams; 4 = Eggs and initial larval stages in natural or constructed basins, after flooding, exotrophic tadpoles in puddles or streams; 5 = Eggs and initial larval stages in constructed underground nests, after flooding, exotrophic tadpoles in wells or streams; 11 = Foam nest floating in lake, exotrophic tadpoles in puddles; 13 = Foam nest floating in accumulated water in constructed basins, exotrophic tadpoles in puddles; 24 = Eggs hatching into exotrophic tadpoles that fall into lentic water; 30 = Foam nest with eggs and initial larval stages in constructed underground nests, after flooding, exotrophic tadpoles in well; Habit: Arboreal; Cryptozoic; Fossorial; Terrestrial and Rheophilic. Type of environment: Le = Lentic; 1 = Temporary puddle; 2 = Permanent puddle; 3 = Wetland; B = Lotic; 1 = Spring; 2 = 1^st^ order stream. Semaphoron: A = Adult; B = Adult and Tadpole. Reference: 1 = Field data (Present study); 2 = Data from biological collections (Bertha Lutz Herpetological Collection, Capão da Imbuia Natural History Museum); 3 = Data from literature (Lutz 1973; Heyer 1979; Heyer 1994; Conte et al. 2009 or Leivas et al. 2018).

Family/Species	Location	Vegetation formation	Population status	Reproductive mode	Habit	Semaphoron	Type of environment	Reference
** Alsodidae **								
*Limnomedusa macroglossa* (Duméril & Bibron, 1841)	FZ	SSF	Descending	1	Rheophlic	B	Le1	1, 2, 3
** Bufonidae **								
*Rhinella diptycha* (Cope, 1862)	FZ	SSF	Stable	1	Terrestrial	A	Le1, Le2	1, 2
*Rhinella ornata* (Spix, 1824)	FZ, CA	SSF/MOF	Stable	1	Terrestrial	A	Lo2	1, 2
** Hylidae **								
*Aplastodiscus perviridis* Lutz, 1950	CA	MOF	Stable	5	Arboreal	A	Le1, Lo2	2
*Boana albopunctata* (Spix, 1824)	FZ, SMI, CA	SSF/MOF	Stable	1	Arboreal	B	Le1, Lo1	1, 2
*Boana caingua* (Carrizo, 1991)	FZ, SMI	SSF	Stable	1	Arboreal	A	Le3	1, 2
Boana cf. caipora	CA	MOF	Stable	2	Arboreal	A	Lo2	2
*Boana curupi* (Garcia, Faivovich & Haddad, 2007)	FZ	SSF	Unknown	2	Arboreal	B	Lo2	1, 2
*Boana faber* (Wied-Neuwied, 1821)	FZ, SMI, CA	SSF/MOF	Stable	4	Arboreal	B	Le3	1, 2, 3
*Boana punctata* (Schneider, 1799)	FZ, SMI	SSF	Stable	1	Arboreal	B	Le2, Le3	1
*Boana raniceps* (Cope, 1862)	FZ, CA	SSF/MOF	Stable	1	Arboreal	A	Le2, Le3	2
*Dendropsophus minutus* (Peters, 1872)	FZ, CA	SSF/MOF	Stable	1	Arboreal	B	Le1, Le2, Lo2	1, 2
*Dendropsophus nanus* (Boulenger, 1889)	FZ	SSF	Stable	1	Arboreal	B	Le1, Le2, Lo2	1, 2, 3
*Itapotihyla langsdorffii* (Duméril & Bibron, 1841)	FZ	SSF	Unknown	1	Arboreal	B	Le1, Lo2	1, 2, 3
*Phyllomedusa tetraploidea* Pombal & Haddad, 1992	CA	MOF	Stable	24	Arboreal	A	Le2, Le3	2
*Ololygon berthae* (Barrio, 1962)	FZ, SMI	SSF	Stable	1	Arboreal	A	Le2, Le3	2, 3
*Scinax fuscovarius* (Lutz, 1925)	FZ, SMI	SSF/MOF	Stable	11	Arboreal	B	Le1, Le2, Lo1	1, 2, 3
*Scinax perereca* Pombal, Haddad & Kasahara, 1995	CA	MOF	Stable	1	Arboreal	A	Le2, Le3	2
*Scinax squalirostris* (Lutz, 1925)	CA	MOF	Stable	1	Arboreal	A	Le2, Le3	2
*Trachycephalus typhonius* (Linnaeus, 1758)	FZ	SSF	Stable	1	Arboreal	B	Le3, Lo2	1, 2, 3
** Hylodidae **								
*Crossodactylus schmidti* Gallardo, 1961	CA	MOF	Descending	3	Rheophlic	A	Lo2	2, 3
** Leptodactylidae **								
*Leptodactylus elenae* Heyer, 1978	FZ	SSF	Stable	30	Terrestrial	B	Le1, Lo2	1, 2
*Leptodactylus fuscus* (Schneider, 1799)	CA	MOF	Stable	30	Terrestrial	A	Le2, Le3	2
*Leptodactylus labyrinthicus*	FZ	SSF	Stable	11	Terrestrial	A	Le2, Le3	3
*Leptodactylus luctator* (Hudson, 1892)	FZ, CA	SSF/MOF	Stable	11	Terrestrial	A	Le2, Le3	2
*Leptodactylus mystacinus* (Burmeister, 1861)	FOZ, SI, CA	SSF/MOF	Stable	30	Terrestrial	B	Le1	1, 2
*Leptodactylus podicipinus* (Cope, 1862)	FZ, SMI	SSF	Stable	13	Criptozóico	B	Le1	1, 2, 3
*Physalaemus carrizorum* Cardozo & Pereyra, 2018	CA	MOF	Stable	11	Terrestrial	A	Le1, Le3	2
*Physalaemus cuvieri* Fitzinger, 1826	FZ, SMI, SI, CA	SSF/MOF	Stable	11	Terrestrial	B	Le1, Le2, Lo2	1, 2
** Microhylidae **								
*Elachistocleis bicolor* (Guérin-Méneville, 1838)	FZ, SMI	SSF	Stable	1	Fossorial	B	Le1, Le2	1, 2
** Odontophrynidae **								
*Proceratophrys avelinoi* Mercadal de Barrio & Barrio, 1993	FZ, SMI, CA	SSF/MOF	Unknown	2	Cryptozoic	B	Lo1, Lo2	1, 2, 3

## ﻿Discussion

We recorded 31 anuran amphibian species in the Iguaçu National Park, representing approximately 20% of the species known for the state of Paraná (Santos-Pereira et al. 2018), 12% of those reported for the Semideciduous Seasonal Forest (SSF), and 4.8% of the species known from the Atlantic Forest domain (Rossa-Feres et al. 2017). This study provides the first species list for amphibians in INP based on systematic surveys, literature records and scientific collections. However, this is not the first study on amphibians conducted in the INP. The earliest records date back to 1973, when Bertha Lutz (1973) reported five anuran species (*Boana faber*, *Dendropsophus nanus*, *Ololygon berthae*, *Scinax fuscovarius*, and *Trachycephalus typhonius*) in the Trilha do Poço Preto in Foz do Iguaçu region. Subsequently, Heyer (1979), used specimens from INP in taxonomic studies of the family Leptodactylidae, and, later, in a revision of the *Leptodactylus podicipinus* group (Heyer 1994), which included *Leptodactylus labyrinthicus* and *Leptodactylus podicipinus* from the INP. After a 15-year gap with no amphibian studies in the park, Conte et al. (2009) published the first record of *Limnomedusa macroglossa* in the state of Paraná, based on specimens collected in INP. More than a decade later, Leivas et al. (2018), reported four additional species occurring in the park: *Crossodactylus schmidti*, *Itapotihyla langsdorffii*, *Proceratophrys bigibbosa*, and *Vitreorana parvula*. Subsequently, the INP Management Plan listed only the 12 previously cited amphibian species with confirmed occurrence in the park (Instituto Chico Mendes de Conservação da Biodiversidade 2018).

In addition to the 31 species recorded in the INP, three others are considered to have probable occurrence in the park: *Vitreorana parvula* (Boulenger, 1895), *Proceratophrys bigibbosa* (Peters, 1872), and *Aquarana catesbeiana* (Shaw, 1802). These species have been reported either in the surroundings of the park (Nazaretti 2016; Leivas et al. 2018) or within INP itself (Nazaretti 2016). However, we did not detect these species during our field surveys, nor did we find voucher specimens in scientific collections. Therefore, although the actual amphibian richness of the INP may exceed the number documented in this study, we adopted a conservative approach in compiling the current species list due to the absence of confirmed records. Regarding the invasive exotic species *A. catesbeiana*, there are records suggesting its presence within the INP, raising concerns about its potential impacts on native species (Hórus Institute for Environmental Conservation and Development 2025). Nevertheless, this record appears to remain controversial, as published accounts restrict the species’ presence to areas adjacent to the INP in neighboring municipalities, without confirmation of its occurrence within the park’s boundaries (Santos-Pereira and Rocha 2015; Leivas et al. 2018). In any case, the possible introduction and spread of this exotic species are concerning, given its documented negative impacts on native amphibian populations through predation on smaller anurans (Oda et al. 2019), competition with native species (Silveira and Guimarães 2021), and the transmission of pathogens such as the chytrid fungus *Batrachochytrium dendrobatidis* and *ranavirus* (Laufer et al. 2017; Ruggeri et al. 2019).

The anuran fauna of INP exhibited nine reproductive modes, corresponding to 33% of the 27 reproductive modes described for the Atlantic Forest (Haddad and Prado 2005). This reproductive diversity exceeds that observed in other areas of SSF and MOF within the Atlantic Forest domain, for example, the eight reproductive modes recorded at Fritz Plaumann State Park (Bastiani and Lucas 2013) and the seven modes documented at Morro do Diabo State Park (Santos et al. 2009). However, the reproductive diversity recorded in PNI remains lower than that found in areas of Dense Ombrophilous Forest, where up to 14 reproductive modes have been reported (e.g., Garey and Hartmann 2012). Two non-mutually exclusive factors may explain the higher reproductive diversity observed in this study compared to other sites. First, there is a positive probabilistic relationship between species richness and reproductive mode diversity, that is, areas with a higher number of species tend to exhibit greater reproductive diversity. Second, precipitation plays an important role, as areas with higher rainfall generally support a broader range of reproductive modes (Vasconcelos et al. 2010). Our study recorded both drought-adapted reproductive modes (e.g., modes 11, 13, and 30, Arzabe 1999; Haddad and Prado 2005; Brassaloti et al. 2010), as well as others (i.e., 5 and 24) typically found in areas with high precipitation (Armstrong and Conte 2010).

Most of the species recorded in the INP are arboreal, followed by terrestrial species, and they primarily use lentic environments for reproduction. These patterns reflect the species richness of Hylidae and Leptodactylidae found in the INP. The predominance of arboreal species is recurrent pattern in anuran communities of the Atlantic Forest (e.g., Brassaloti et al. 2010; Vicente-Ferreira et al. 2024; Souza-Costa et al. 2024). Similarly, the predominance of species in lentic environments has also been recorded for the Atlantic Forest (e.g., Jordani et al. 2017). This pattern is associated with a greater diversity of reproductive modes found in lentic environments (Haddad and Prado 2005) and, possibly, with the deposition of eggs in lentic environments and exotrophic tadpoles being the basal condition of reproductive modes in anurans (Gomez-Mestre et al. 2012).

According to IUCN data (2024), the majority of species recorded in INP have stable populations. Three species (*Boana curupi*, *Itapotihyla langsdorffii*, and *Proceratophrys avelinoi*) have an unknown population status, highlighting the need for monitoring these populations, across Atlantic Forest. Two species, *Crossodactylus schmidti* and *Limnomedusa macroglossa*, exhibit declining populations. Both species occur in lotic environments within forest interiors (Toledo et al. 2021; Garey et al. 2024). These population declines are mainly associated with habitat modification, pollution, and agricultural activities (IUCN 2024). We therefore recommend long-term monitoring of these two species’ populations in the INP, which represents the largest forest fragment of the inland Atlantic Forest.

The presence of *Boana curupi* in the INP represents the first record of this species in the state of Paraná. This species of treefrog occurs in northeastern Argentina (Misiones Province; Frost 2024), southeastern Paraguay (Caazapá, Guairá, and Itapúa Departments, Weiler et al. 2013), and southern Brazil (western Santa Catarina and northeastern/northern Rio Grande do Sul, Frost 2024). Our recorded in the INP extends the known geographic distribution of *B. curupi* by approximately 100 km northwestward. We recorded *Boana curupi* in areas of SSF areas, calling on riparian vegetation along lotic water bodies where we also collected tadpoles. We also recorded *Physalaemus carrizorum*, confirming the first record of this species in the state of Paraná. This species is known from Misiones Province in Argentina, Paraguay, and the state of Rio Grande do Sul in Brazil (Cardozo and Pereyra 2018; Potrich et al. 2020; Netto and Brusquetti 2023; Frost 2024). The INP record extends its geographic range of *P. carrizorum* by ca 202 km to the northeast. Additionally, we recorded the presence of Boana cf. caipora in INP. Due to significant morphological variability, the absence of molecular and bioacoustic data, and the fact that *B. caipora* has only been recorded from the Serra do Mar in the state of São Paulo (Frost 2024), more than 800 km away, we opted for a conservative identification, indicating taxonomic uncertainty. However, if future studies confirm these specimens as *Boana caipora*, this would represent the first record of the species in the state of Paraná. Further research involving more adult individuals and the inclusion of tadpole, bioacoustic, and genetic data will be essential to confirm the species’ identity.

The anuran species composition in the INP closely resembles that of Iguazú National Park in Argentina, likely because both parks form part of the same Atlantic Forest fragment, separated only by the Iguaçu River. In the Iguazú National Park (Argentina), researchers have recorded 32 anuran species (López and Garey 2021), with 24 species shared between the Iguazú and Iguaçu National Parks. However, eight species (*Aquarana catesbeiana*, *Dendropsophus sanborni*, *Melanophryniscus devincenzii*, *Ololygon aromothyella*, *Odontophrynus americanus*, *Scinax nasicus*, *Rhinella icterica*, and *Vitreorana parvula*) have been recorded only on the Argentine side, while six species (*Aplastodiscus perviridis*, *Phyllomedusa tetraploidea*, *Crossodactylus schmidti*, Boana cf. caipora, *Boana punctata*, and *Boana curupi*) have been recorded only on Brazilian side. Consequently, this largest inland forest fragment of the Atlantic Forest harbors a total 38 anuran species, highlighting the importance of this remnant for the conservation of amphibian biodiversity in South America.

## ﻿Conclusion

We establish the first systematic species list for Iguaçu National Park amphibian assemblage, providing data for this UNESCO World Heritage Site’s. The Iguaçu National Park, together with the Iguazú National Park in Argentina, forms the largest remaining inland fragment of the Atlantic Forest, harboring 38 amphibian species; more than those recorded in other forest fragments of the same vegetation type (e.g., 37 species in Uetanabaro et al. 2007; 34 species in Brassaloti et al.,2010; 19 species in Vasconcelos et al. 2011) however, fewer than the 46 species observed by Souza-Costa et al. (2024). These findings highlight Iguaçu National Park’s dual conservation significance: as a refuge for amphibian biodiversity and as a potential source population for regional recolonization of other forest fragments. We emphasize the urgent need for implementing comprehensive monitoring programs and science-based management strategies to safeguard this amphibian biodiversity and its ecosystem functions.

## ﻿Additional information

### Use of AI

No use of AI was reported.

### Author ORCIDs

Rodrigo Lingnau https://orcid.org/0000-0001-8406-4439

Michel V. Garey https://orcid.org/0000-0002-7482-792X

### Data availability

All of the data that support the findings of this study are available in the main text or Supplementary Information.

## Supplementary materials

Supplementary material 1 List of specimens Data type docx Explanation note List of specimens collected in the Iguaçu National Park, deposited in the Bertha Lutz Herpetological Collection at the Federal University of Latin American Integration – UNILA, Foz do Iguaçu, Paraná, Brazil. https://binary.pensoft.net/file/1492627This dataset is made available under the Open Database License (http://opendatacommons.org/licenses/odbl/1.0/). The Open Database License (ODbL) is a license agreement intended to allow users to freely share, modify, and use this Dataset while maintaining this same freedom for others, provided that the original source and author(s) are credited.Emerson A. da Rosa, Rodrigo Lingnau, Michel V. Garey
