# Supplementary material for: ﻿Amphibians of the largest inland Atlantic Forest fragment, Iguaçu National Park, Paraná State, southern Brazil
Source: Zookeys. 2025 Dec 15;1264:109–28. doi: 10.3897/zookeys.1264.164796 (PMC12723401; doi:10.3897/zookeys.1264.164796)
Supplement: Supplementary material 1 — List of specimens [file zookeys-1264-109_article-164796__-s001.docx]

**Supplementary Material 1.** List of Specimens Collected in the Iguaçu National Park, deposited in the Bertha Lutz Herpetological Collection at the Federal University of Latin American Integration – UNILA, Foz do Iguaçu, Paraná, Brazil.

**Adults Anurans**: **Alsodidade.** ***Limnomedusa macroglossa*** (CA 0970; CA 2024); **Bufonidae. *Rhinella diptycha*** (CA 0962; CA 0963; CA 0994); ***Rhinella ornata*** (CA 1883; CA 1891 CA 2030); **Hylidae. *Aplastodiscus perviridis*** (CA 007); ***Boana albopunctata*** (CA 012; CA 1042; CA 2021; CA 2022); ***Boana caingua*** (CA 1069; CA 2027; CA 2028; CA 2029); ***Boana curupi*** (CA 2011; CA 2012; CA 2013; CA 2014; CA 2015) ***Boana faber*** (CA 006; CA 2023; CA 2024; CA 2025; CA 2026) ***Boana punctata*** (CA 1069; CA 006; CA 1079; CA 1080; CA 1081; CA 1082; CA 1088; CA 1089; CA 1090; CA 1091; CA 1092) ***Boana raniceps*** (CA 1062; CA 0965); ***Dendropsophus minutus*** (CA 0953; CA 0954; CA 0955; CA 0956; CA 0957; CA 0958; CA 0959; CA 0960; CA 0961); ***Dendropsophus nanus*** (CA 001; CA 002; CA 1057; CA 1058; CA 1059; CA 1060; CA 1061; CA 1066; CA 1067; CA 1075; CA 1076; CA 1077; CA 1078; CA 1083; CA 1084; CA 1085; CA 1086); ***Itapotihyla langsdorffi*** (CA 048; CA 049; CA 968; CA 969) ***Scinax fuscuvarius*** (CA 014; CA 0977; CA 0978; CA 0979; 044; CA 0980; CA 0981; CA 0982; CA 0983; CA 0984; CA 0985; CA 0986; CA 0987; CA 0998; CA 0999; CA 1000; CA 1001; CA 1002; CA 1003; CA 1043; CA 1044; CA 1045; CA 1046; CA 1047; CA 1048; CA 1049; CA 1050; CA 1051; CA 1053; CA 1054; CA 1055; CA 1056; CA 1073; CA 1074) ***Ololygon berthae*** (CA 1034; CA 1035; CA 1036) ***Trachycephalus typhonius*** (CA 061; CA 2036; CA 2037); **Hylodidae. *Crossodactylus schimidti*** (CA 003; CA 004; CA 005; CA 009; CA 010; CA 011; CA 015; CA - 1902); **Leptodactylidae. *Leptodactylus elenae*** (CA 1063; CA 1064; CA 2031; CA 2032; CA 2033) ***Leptodactylus podicipinus*** (CA 046; CA 047; CA 0946; CA 0947; CA 0948; CA 0949; CA 1039; CA 1040; CA 1041; CA 1071; CA 1087); ***Leptodactylus luctator*** (CA – 045; CA 1072) ***Leptodactylus mystacinus*** (CA – 1900) ***Physalaemus cuvieri*** (CA 1042; CA 1893; CA 1894; CA 1895; CA 1896; CA 1897; CA 1898; CA 1899; CA 1906; CA 1907; CA 1908; CA 1909; CA 1911; CA 1912; CA 1913; CA 1914; CA 1915; CA 2003; CA 2004; CA 2005; CA 2006); **Microhylidae. *Elachistocleis bicolor*** (CA 1052; CA 1065; CA 1089; CA 2007; CA 2008; CA 2009); **Odontophrynidae. *Proceratophrys avelinoi*** (CA 013; CA 1892; CA 1901; CA 1904; CA 1905; CA 1910; CA 2016; CA 2017; CA 2018; CA 2019; CA 2020).

**Tadpoles: Alsodidae. *Limnomedusa macroglossa* (**CA G476); **Hylidae. *Boana albopunctata*** (CA G466); ***Boana curupi*** (CA G492; CA G496; CA G497; CA G498; CA G500). ***Boana faber* (**CA G475; CA G482; CA G494; CA G503). ***Boana punctata*** (CA G495; CA G499). ***Dendropsophus minutus*** (CA G467; CA G472; CA-G485; CA-G487; CA-G488; CA-G491). ***Dendropsophus nanus*** (CA G477; CA G480; CA G483). ***Itapotihyla langsdorffi*** (CA G464). ***Scinax fuscuvarius*** (CA G463; CA G465; CA G496; CA G470; CA G471; CA G478; CA G479; CA G486; CA G489; CA G501; CA G502). ***Trachycephalus typhonius* (**CA G474**); Leptodactylidae*. Leptodactylus elenae*** (CA G490); ***Leptodactylus podicipinus*** (CA G473; CA-G493); ***Leptodactylus mystacinus*** (CA-G505); ***Physalaemus cuvieri*** (CA G461; CA G462; CA G504). **Microhylidae**. ***Elachistocleis bicolor*** (CA G468). **Odontophrynidae. *Proceratophrys avelinoi*** (CA G484).

List of specimens collected in the Iguaçu National Park, deposited in the Herpetological Collection of the Capão da Imbuia Natural History Museum, Curitiba, Paraná, Brazil.

**Adults Anurans:** **Alsodidae**. ***Limnomedusa macroglossa*** (MHNCI 10798; MHNCI 10801; MHNCI 10802; MHNCI 10825; MHNCI 10826; MHNCI 10829); **Bufonidae**. ***Rhinella diptycha*** (MHCNI 10783) ***Rhinella ornata*** (MHNCI 10819; MHNCI 10820; MHNCI 10821; MHNCI 10822; MHNCI 10866); **Hylidae**. ***Aplastodiscus perviridis*** (MHNCI 10814; MHNCI 10815; MHNCI 10816; MHNCI 10817; MHNCI 10818) ***Boana albopunctata*** (MHNCI 10790; MHNCI 10791; MHNCI 10792; MHNCI 10793) ***Boana caingua*** (MHNCI 10837; MHNCI 10838; MHNCI 10839; MHNCI 10840) ***Boana*** **cf. *caipora*** (MHNCI 10834; MHNCI 10835; MHNCI 10836) ***Boana curupi*** (MHNCI 10824) ***Boana faber*** (MHNCI 10788; MHNCI 10789) ***Boana raniceps*** (MHNCI 10787) ***Dendropsophus minutus*** (MHNCI 10843; MHNCI 10844; MHNCI 10845; MHNCI 10846; MHNCI 10847; MHNCI 10848) ***Dendropsophus nanus*** (MHNCI 10841; MHNCI 10842) ***Itapotihyla langsdorffii*** (MHNCI 10784; MHNCI 10785; MHNCI 10786) ***Phyllomedusa tetraploidea*** (MHNCI 10862; MHNCI 10863; MHNCI 10864; MHNCI 10865) ***Scinax fuscuvarius*** (MHNCI 10796; MHNCI 10799) ***Scinax perereca*** (MHCI 10823) ***Scinax squalirostris*** (MHNCI 10796; MHNCI 10799; MHNCI 10849; MHNCI 10850; MHNCI 10851; MHNCI 10852; MHNCI 10853) **Hylodidae. *Crossodactylus schimidti*** (MHNCI 10807; MHNCI 10827; MHNCI 10831; MHNCI 10832); **Leptodactylidae. *Leptodactylus elenae*** (MHNCI 10867) ***Leptodactylus fuscus*** (MHNCI 10813) ***Leptodactylus luctator*** (MHNCI 10794; MHNCI 10795) ***Leptodactylus mystacinus*** (MHNCI 10797) ***Leptodactylus podicipinus*** (MHNCI 10803; MHNCI 10804; MHNCI 10805; MHNCI 10808) ***Physalaemus* aff. *gracillis*** (MHNCI 10806; MHNCI 10812; MHNCI 10830) ***Physalaemus cuvieri*** (MHNCI 10809; MHNCI 10811; MHNCI 10828; MHNCI 10833); **Microhylidae. *Elachistocleis bicolor*** (MHNCI 10854); **Odontophrynidae. *Proceratophrys avelinoi*** (MHNCI 10855; MHNCI 10856; MHNCI 10857; MHNCI 10858; MHNCI 10859; MHNCI 10860; MHNCI 10861.
